# Suicidal attempt with caffeine overdose treated with dexmedetomidine: a case report

**DOI:** 10.1186/s13256-020-02611-6

**Published:** 2021-01-16

**Authors:** Teppei Kitano, Masaki Okajima, Koji Sato, Toru Noda, Takumi Taniguchi

**Affiliations:** Intensive Care Unit, Kanazawa University Hospital, Kanazawa, 920-8641 Japan

**Keywords:** Caffeine, Dexmedetomidine, Overdose, Poisoning, Suicide

## Abstract

**Background:**

Caffeine is a widely used dietary stimulant, and cases of caffeine overdoses, sometimes leading to death, are increasing. We encountered a case of caffeine intoxication resolved with administration of the sedative agent dexmedetomidine.

**Case presentation:**

We administered dexmedetomidine for sedation and to suppress sympathetic nerve stimulation in the case of an 18-year-old Japanese male who ingested a massive dose of caffeine with the intention of committing suicide. The patient was in an excited state and had hypertension, sinus tachycardia, and hypokalemia with prominent QT prolongation. After dexmedetomidine administration, the patient’s mental state, hemodynamics, and electrolyte levels were improved immediately. He was discharged without any sequelae 3 days later.

**Conclusion:**

Cases of acute caffeine intoxication with agitation, sympathetic overactivity and adverse cardiac events would benefit with dexmedetomidine treatment.

## Background

Caffeine is ingested through food and drink daily and overdoses have become more likely due to the recent spread of energy drinks and sleepiness-prevention medicines [1]. Death from caffeine intoxication in Japan was first reported in 2015, and there is concern that its incidence will increase [2]. Kamijo *et al.* reported 3 deaths out of 7 cardiopulmonary arrest among 101 caffeine intoxication patients in Japan [1]. We encountered a case of caffeine intoxication resolved with administration of the sedative agent dexmedetomidine. It is considered that dexmedetomidine is effective against caffeine intoxication because of its pharmacological action [3].

## Case presentation

**Fig. 1:**
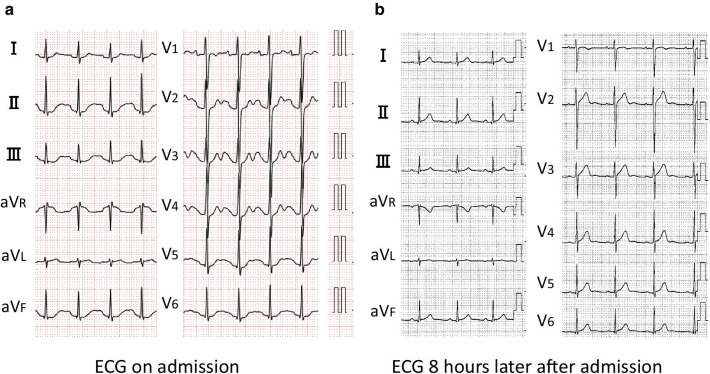
Electrocardiography. **a** Electrocardiography revealed prominent QT prolongation on admission. **b** Eight hours later, QT prolongation was improved.

**Fig. 2:**
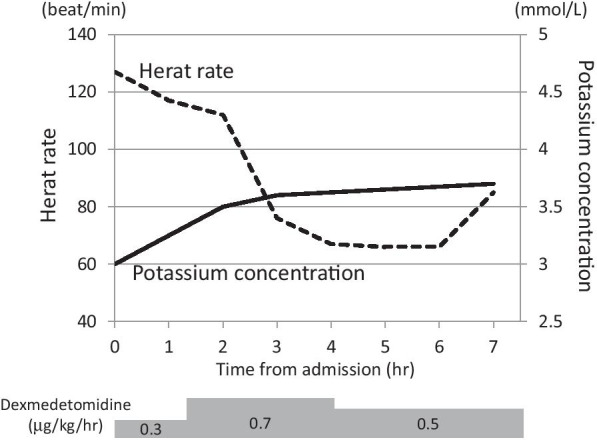
Time course of heart rate and potassium concentration during dexmedetomidine administration.

An 18-year-old healthy non-smoking Japanese male ingested 60 tablets of sleepiness-prevention medicine (containing a total of approximately 6.0 g caffeine) in a suicide attempt. Eighty minutes later he was transferred to the emergency department of our hospital because of complaints of nausea and headache. In his past social history, he got maladjustment to high school since he hit his friend and was suspended from school for ten days by principal. Although no personal and family history of psychiatric illness was noted, a scar of wrist cutting was observed. On arrival, his vital signs included Glasgow Coma Scale of 15, his blood pressure of 152/82 mmHg, pulse rate of 139 beats/min, respiratory rate of 22/min, oximetry of 100% on room air, and body temperature of 36.8 °C. Pertinent findings on physical examination included body weight of 58.8 kg, clear breath sounds and a regular, rapid heart rhythm with systolic ejection murmur on auscultation. Electrocardiogram showed sinus tachycardia at a rate of 139 beats/min with QT prolongation (QTc 617 ms) (Fig. 1a). Arterial blood gas analysis showed lactic acidosis (lactate 3.8 mmol/L). The other laboratory data were as follows: white blood cells 11,120/mcL; hemoglobin 17.4 g/dL; platelets 24.5×10^4^/mcL; blood urea nitrogen 13 mg/dL (4.6 mmol/L); serum creatinine 0.73 mg/dL (64.5 mcmol/L); sodium 142 mmol/L; potassium 2.6 mmol/L; chloride 103 mmol/L; creatinine phosphokinase 126 IU/L (Table 1). We observed nothing special on systemic computed tomography (CT) scan. After arrival, we performed gastric lavage of 1600 mL and administered 30 g of activated charcoal through a nasogastric tube. We administered total amount of 20 mmol potassium at the rate of 20 mmol/hour, and the patient was admitted to an intensive care unit. As he was agitated, with a Richmond Agitation-Sedation Scale score of + 2 as a symptom of acute caffeine poisoning, we immediately started administration of dexmedetomidine (maximum 0.7 µg/kg/hour) (Fig. 2). Seven hours later, serum potassium and lactate normalized to 3.7 mmol/L and 1 mmol/L, respectively, and QTc also normalized (Fig. 1b). Administration of dexmedetomidine was stopped at 12 hours after dexmedetomidine infusion because he got quiet. Because he still had suicide ideation, he was hospitalized in psychiatric ward for medical protection. He said he just has been thinking of dying since high school and did not talk anymore. He was gradually free from suicide ideation and was discharged on his third day in hospital.

**Table 1:** Laboratory data

Laboratory test	Results	Reference range
White blood cell count	11.12 × 10^9^ /L	3.3–8.8 × 10^9^ /L
Red blood cell count	5.56 × 10^12^ /L	4.3–5.5 × 10^12^ /L
Hemoglobin	17.4 g/dL	13.5–17.0 g/dL
Hematocrit	46.9 %	39.7–51.0 %
Platelet count	245 × 10^9^ /L	130–350 × 10^9^ /L
Protrombin-INR	1.10	0.91–1.14
Activated partial thromboplastin time	27.6 s	26.9–38.1 s
D-dimer	0.4 mg/mL	0–1.0 mg/mL
Sodium	142 mmol/L	135–149 mmol/L
Potassium	2.6 mmol/L	3.5–4.9 mmol/L
Chloride	103 mmol/L	96–108 mmol/L
Magnesium	2.0 mg/dL	1.6–2.4 mmol/L
Asparate aminotransferase	21 IU/L	13–33 IU/L
Alanine aminotransferase	19 IU/L	8–42 IU/L
Lactate dehydrogenase	192 IU/L	119–229 IU/L
Blood urea nitrogen	13 mg/dL	8.0–22.0 mg/dL
Creatinine	0.73 mg/dL	0.60–1.00 mg/dL
Creatininekinase	126 IU/L	62–287 IU/L
Amylase	61 IU/L	40–113 IU/L
Total bilirubin	0.9 mg/dL	0.3–1.2 mg/dL
Total protein	8.2 mg/dL	6.7–8.3 mg/dL
Glucose	145 mg/dL	69–109 mg/dL
Troponin T	0.004 ng/mL	0–0.014 ng/mL
pH	7.41	7.36–7.44
Partial pressure of arterial oxygen	97.8 Torr	75–100 Torr
Partial pressure of arterial carbon dioxide	33.9 Torr	36–44 Torr
Bicarbonate	21.1 mmol/L	22–26 mmol/L
Lactate	3.6 mmol/L	0.56–1.39 mmol/L

*INR* international normal ratio, *IU* international unit

## Discussion and conclusions

Caffeine is present in drinks such as coffee, tea, energy drinks, and is ingested as part of the daily diet [4]. Caffeine is member of a class of drugs known as methylxanthines and has effects of drowsiness prevention, cardiac inotropic effect, and diuresis by antagonizing adenosine receptors [5]. While higher intake of caffeine led lower suicide risk with its pharmacological antidepressant effect in daily situations, overdoses of caffeine cause potentially lethality [6, 7]. Caffeine is rapidly and completely absorbed orally, and the blood concentration of caffeine reaches a maximum level after 30–120 minutes. Its elimination half-life ranges from 3 hours in smokers to 10 hours in non-smokers and can be longer after overdose [8]. Caffeine is metabolized mainly by CYP1A2, expressed in the liver, and is excreted in urine as uric acid. It is reported that caffeine may lead to acute intoxication with ingestion of 15–30 mg/kg or more, and death with ingestion of 100–200 mg/kg or more [9]. In this case, the patient is a non-smoker and caffeine concentration is estimated to have reached approximately maximum level when he was transferred to our hospital 80 minutes after oral ingestion. Moreover, estimated caffeine ingestion was 100 mg/kg or more which means he was in a critical situation with a high likelihood of seizures and life-threatening arrhythmias. Indeed, in this patient, QT prolongation was observed with hypokalemia which indicated that lethal arrhythmias such as ventricular tachycardia and ventricular fibrillation were more likely to occur. The mechanism of hypokalemia caused by caffeine intoxication is still unclear. Caffeine has been reported to act as an activator of the sympathetic activator in central nervous system [10]. Moreover, caffeine also has phosphodiesterase inhibitor activity and inhibits the degradation of cyclic adenosine monophosphate (cAMP) produced by beta receptor-adenyl cyclase followed by augmentation of beta adrenergic action.

Taken together, caffeine augments beta adrenergic action by both the stimulation of sympathetic nerves in the brain and augmentation of beta action in peripheral tissues such as myocardium. Beta stimulation shifts potassium from extracellular areas to intracellular areas. Dexmedetomidine exerts a sympatholytic effect in the brain by decreasing cAMP. Therefore, in this case, the dexmedetomidine we administered inhibited beta action which led to restoration of potassium distribution. This could explain why potassium concentration was normalized by only 20 mmol potassium administration, much less than the estimated potassium deficiency. Additionally, dexmedetomidine normalized both high blood pressure and high heart rate by inhibition of the activated sympathetic nervous system caused by caffeine poisoning. Beta blockers are also sympatholytic drugs and have been used in patients with caffeine intoxication. Beta blockers have an antiadrenergic effect in downstream organs such as the heart and peripheral arteries. On the other hand, dexmedetomidine directly acts on the central nervous system as an upstream sympatholytic agent. Therefore, it is reasonable to use dexmedetomidine to supplement beta blockers in patients with caffeine intoxication. Dexmedetomidine might be an additional therapeutic option to avoid adverse cardiac events in patients with caffeine intoxication. We experienced a case of acute caffeine intoxication with agitation and sympathetic overactivity, which was improved immediately by administration of dexmedetomidine.

## Data Availability

The dataset used in this case report is available from the corresponding author on reasonable request.

## Abbreviation

cAMP: Cyclic adenosine monophosphate
